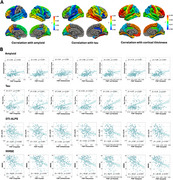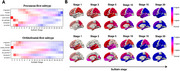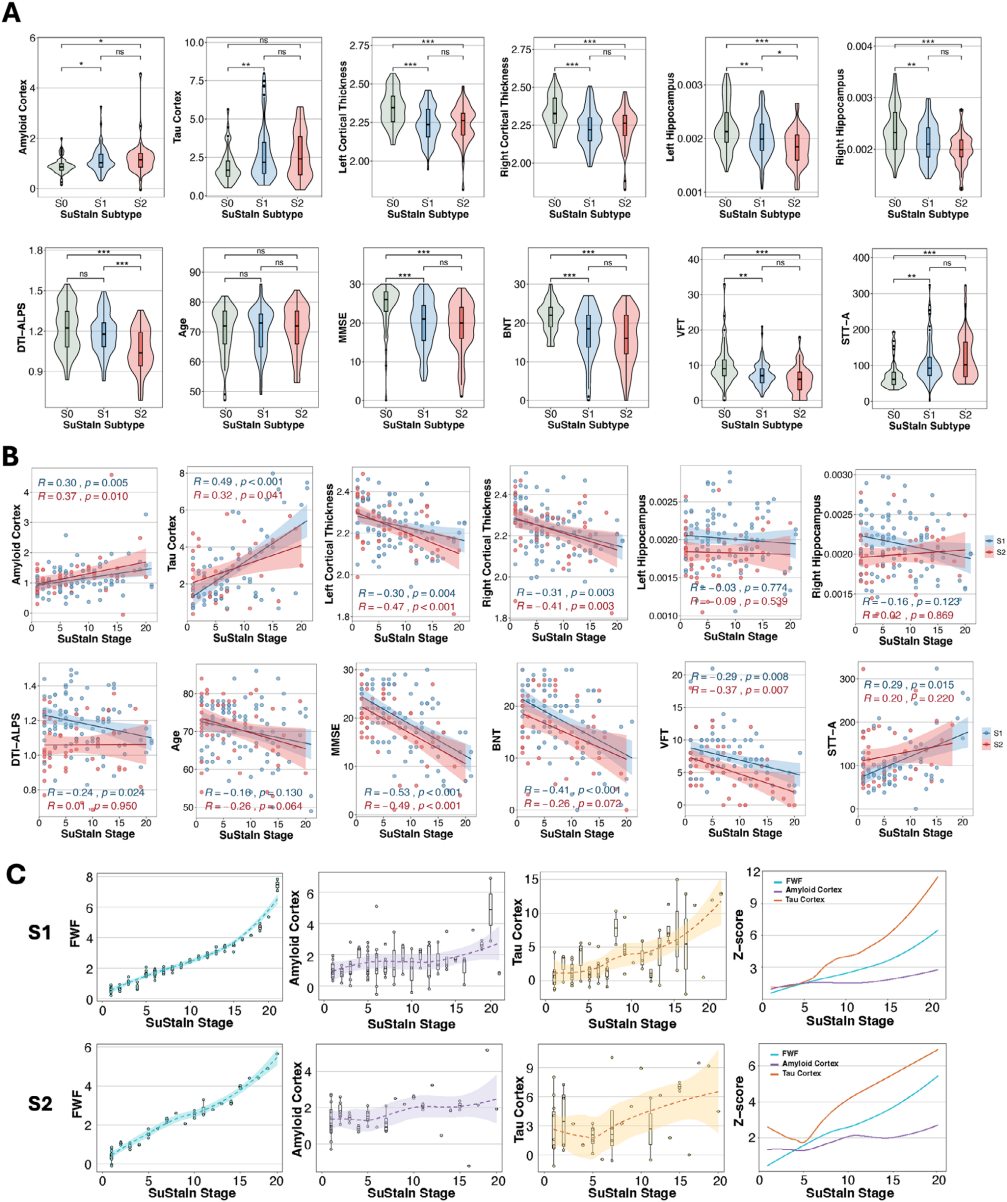# Distinct spatiotemporal patterns of juxtacortical microstructure in Alzheimer's Disease

**DOI:** 10.1002/alz70856_097784

**Published:** 2025-12-24

**Authors:** Binyin Li, Junfang Zhang

**Affiliations:** ^1^ Ruijin Hospital affiliated to Shanghai Jiaotong University School of Medicine, Shanghai, Shanghai, China; ^2^ Shanghai Sixth People's Hospital Affiliated to Shanghai Jiao Tong University School of Medicine, Shanghai, Shanghai, China

## Abstract

**Background:**

Alzheimer's disease (AD) exhibits heterogeneity in pathological changes involving β‐amyloid (Aβ), tau, neural macrostructure and microstructure. We aimed to evaluate juxtacortical free water fraction (FWF), its correlation with neuropathological severity, and its spatiotemporal pattern using MRI and PET in AD.

**Method:**

This prospective study included 198 AD participants with Aβ PET positivity (mean age, 70.6 years ± 7.7 [SD]; 120 women) and 161 cognitively normal participants (CN) (mean age, 66.1 years ± 8.6; 103 women). Diffusion MRI was used to measure FWF and diffusion tensor image analysis along the perivascular space (DTI‐ALPS) index. Cortical thickness and hippocampus volume were automatically segmented using T1‐weighted sequences. The ^18^F‐Florbetapir and ^18^F‐MK‐6240 were used to visualize Aβ and tau accumulation separately. The machine‐learning algorithm Subtype and Stage Inference (SuStaIn) was employed to model the spatiotemporal patterns of juxtacortical FWF change.

**Result:**

When compared to CN, we observed an extensive increase of FWF in AD (all *p* < 0.05), which correlated with Aβ and tau deposition, as well as cortical thinning (all *p* < 0.05, Figure 1A and 1B). Using SuStaIn, we identified two distinct spatiotemporal trajectories of FWF changes (Figure 2A and 2B). The Orbitofrontal‐first subtype had smaller left hippocampus volume (*p* = 0.02) and lower DTI‐ALPS index (*p* < 0.001) compared to the Precuneus‐first subtype (Figure 3A). There were significant correlations between SuStaIn stages and amyloid and tau deposition in the cortex, as well as cortical thickness (all *p* <0.05). The significant negative correlation between SuStaIn stages and DTI‐ALPS index was only found in the Precuneus‐first subtype (*r* = ‐0.24, *p* = 0.024). Increased SuStaIn stage was associated with worse cognitive performance assessed by Mini‐Mental State Examination (MMSE) in both subtypes (all *p* < 0.01, Figure 3B). Figure 3C displays inferred trajectories of FWF, amyloid and tau deposition in the cortex across SuStaIn stages in the two different subtypes. Visually comparing the biomarkers showed that the amyloid deposition in the cortex was found to reach a plateau early than FWF and tau.

**Conclusion:**

Juxtacortical FWF change exhibited diverse spatiotemporal patterns during AD progression, which emerged as a coexisting pathophysiological feature alongside amyloid deposition in the pathogenesis of AD.